# Apoptotic p53 Gene Expression in the Regulation of Persistent Organic Pollutant (POP)-Induced Oxidative Stress in the Intertidal Crab *Macrophthalmus* *japonicus*

**DOI:** 10.3390/antiox11040771

**Published:** 2022-04-13

**Authors:** Kiyun Park, Ihn-Sil Kwak

**Affiliations:** 1 Fisheries Science Institute, Chonnam National University, Yeosu 59626, Korea; kiyunpark@chonnam.ac.kr; 2 Department of Ocean Integrated Science, Chonnam National University, Yeosu 59626, Korea

**Keywords:** crustacean, hexabromocyclododecanes (HBCDs), 2,2′,4,4′-tetrabromodiphenyl ether (BDE-47), apoptosis, oxidative stress, *Macrophthalmus japonicus*, crabs

## Abstract

Persistent organic pollutants (POPs), some of the most dangerous chemicals released into the aquatic environment, are distributed worldwide due to their environmental persistence and bioaccumulation. In the study, we investigated *p53*-related apoptotic responses to POPs such as hexabromocyclododecanes (HBCDs) or 2,2′,4,4′-tetrabromodiphenyl ether (BDE-47) in the mud crab *Macrophthalmus japonicus*. To do so, we characterized *M. japonicus* *p53* and evaluated basal levels of *p53* expression in different tissues. *M. japonicus* *p53* has conserved amino acid residues involving sites for protein dimerization and DNA and zinc binding. In phylogenetic analysis, the homology of the deduced *p53* amino acid sequence was not high (67–70%) among crabs, although *M. japonicus* *p53* formed a cluster with one clade with *p53* homologs from other crabs. Tissue distribution patterns revealed that the highest expression of *p53* mRNA transcripts was in the hepatopancreas of *M. japonicus* crabs. Exposure to POPs induced antioxidant defenses to modulate oxidative stress through the upregulation of catalase expression. Furthermore, *p53* expression was generally upregulated in the hepatopancreas and gills of *M. japonicus* after exposure to most concentrations of HBCD or BDE-47 for all exposure periods. In hepatopancreas tissue, significant increases in *p53* transcript levels were observed as long-lasting apoptotic responses involving cellular defenses until day 7 of relative long-term exposure. The findings in this study suggest that exposure to POPs such as HBCD or BDE-47 may trigger the induction of cellular defense processes against oxidative stress, including DNA repair, cell cycle arrest, and apoptosis through the transcriptional upregulation of *p53* expression in *M. japonicus*.

## 1. Introduction

Persistent organic pollutants (POPs), hexabromocyclododecanes (HBCDs) and 2,2′,4,4′-tetrabromodiphenyl ether (BDE-47), were frequently detected in marine environments because of their wide application as brominated flame retardants in polyurethane plastics, electrical appliances, and expanded or extruded polystyrene foam in buildings, textiles, and vehicles for thermal insulation [1,2]. Due to their persistence, long-range transportability, and bioaccumulation, POPs such as HBCD and BDE-47 have become regarded as priority pollutants, raising increased attention regarding their potential adverse effects on the environment and living organisms, including humans [3,4]. BDE-47, the most prevalent congener, is highly concentrated in the marine environment because of its water solubility and volatility and induces developmental toxicity in benthic organisms including fish [5,6,7]. HBCD is a brominated flame retardant that is used worldwide in expanded and extruded polystyrene foam. HBCD can easily accumulate in animals and humans and cause neurotoxicity, thyroid hormone disruption, and reproductive disorders [8,9]. Potential toxic risks to benthic invertebrates as well as the marine environments involving these pollutants have been emerging despite being globally banned because of their characteristics as POPs. However, there are insufficient studies concerning the adverse effects of POPs such as BDE-47 or HBCD on crustaceans [9,10].

Apoptosis is a normal physiological mechanism that assists in maintaining the balance of cellular homeostasis [11]. The tumor suppressor gene, *p53*, is a key coordinator of cellular homeotic responses to stress signals [11]. It plays critical functions in apoptosis, cell cycle arrest, and DNA repair [12]. BDE-47 exposure induces oxidative stress through the inhibition of the activation of an antioxidant, c-Jun N-terminal kinase, which can mitigate apoptosis in zebrafish embryos [6]. BDE-47 also causes remarkable oxidative damage to cells of *Lemna* *minor* [13]. HBCD exposure produces oxidative stress and the induction of apoptosis through the regulation of genes related to cell apoptosis involving caspases and *p53* in zebrafish embryos [14]. In addition, apoptosis, oxidative stress, and the suppression of protein synthesis are induced by environmentally realistic concentrations of HBCD in marine medaka (*Oryzias melastigma*) fish embryos [15].

Brominated flame retardant pollutants are transported both in solution and attached to suspended particulate matter from continental erosion to the oceans [2]. During transport, permanent or temporary storage takes place in the sediments of estuaries and coastal waters. Marine sediments are a major source of contaminants and are generally considered to behave as a sink for pollutants such as POPs, as well as heavy metals, in aquatic environments [16]. The mud crab *Macrophthalmus japonicus*, as a dominant bioturbator, represents an ecosystem engineer that digs burrows which can trap sediments. This can support the turnover of environmental physical and chemical habitats and the microbial communities living within it [17]. *M. japonicus* crab burrows increase the sediment surface area and provide greater potential for oxygen diffusion and the transition of environmental chemical properties [18]. There is limited information regarding transcriptional responses of the apoptosis-related *p53* gene to HBCD and BDE-47 toxicity on marine invertebrates. The induction of *p53* expression is observed in the intertidal copepod *Tigriopus japonicus* exposed to endocrine-disrupting chemicals or HBCD [19,20]. *p53* has important functions during spermiogenesis in the Chinese mitten crab *Eriocheir sinensis* [12].

In this study, we evaluated oxidative stress and cellular damage in the gills and hepatopancreas of crabs to exposure of POPs, which have a persistence and bioaccumulation in the aquatic ecosystem. To do this, we investigated the potential transcriptional effects of *p53*-related apoptosis and *catalase*-associated antioxidation on *M japonicus* mud crabs after exposure to POPs such as HBCD and BDE-47.

## 2. Materials and Methods

### 2.1. Organisms and Exposure Experiments

Healthy *M. japonicus* crabs (body weight: 9 ± 1.5 g), purchased from a local fish market in Yeosu city (Jeonnam, Korea), were maintained in glass containers (45.7 × 35.6 × 30.5 cm). The environmental conditions were supplemented with a continuous flow of aerated, contaminant-free seawater, as described previously by Park et al. [21]. Before beginning experiments, the crabs were acclimated for 1 week under laboratory conditions with 25% salinity, 20 °C, and a 12 h light–dark period. The crabs were fed small amounts (~200 mg) of TetraMin (Tetra-Werke, Melle, Germany) every day. All experimental procedures were conducted in accordance with the guidelines of the Chonnam National University (Yeosu, South Korea) Institutional Animal Care and Use Committee. The date of approval for the animal experiment was 20 October 2019 (ethical code: CNUIACUC-YS-2019-7C).

HBCD and BDE-47 were purchased from Sigma-Aldrich (St. Louis, MO, USA) and AccuStandard (New Haven, CT, USA) and were of analytical grade. They were dissolved in dimethyl sulfoxide (DMSO; >99.9%; Sigma-Aldrich) to produce stock solutions. The dose of control was <0.01% DMSO. In POPs exposures, 10 crabs were used for treatment with each dose of three nominal concentrations (1, 10, and 100 µg L^−1^ for HBCD; 1, 10, and 30 µg L^−1^ for BDE-47) of each chemical for an exposure period of 1 d, 4 d, and 7 d. All experiments were performed in seawater changed three times every day. 

### 2.2. Macrophthalmus Japonicus p53 (Mjp53) 

A *p53* nucleotide sequence was isolated from the database of 454 GS FLX *M. japonicus* transcriptome [22]. Similarities of *Mjp53* with other *p53* and *p53*-like proteins in crabs were analyzed using the NCBI BLAST program. The ClustalW2 and GeneDoc (v2.6.001) programs were used for multiple alignments and display of *p53* sequences. We used the ProtTest program (v.4.1.5) for determination of a good model of amino acid substitutions and the Gblocks program (v.0.91b) for selection of conserved sequence blocks. A phylogenic relationship was analyzed with the 22 deduced amino sequences (159 aa) of *p53*-related genes using MegaX program (v.10.04). Bootstrap value was 1000 replicates.

### 2.3. Basal Levels of Mjp53 by Tissue and Expression Analysis of Mjp53 Gene

For investigating basal levels of *Mjp53* expression in various tissues, total RNA was extracted from various tissues (hepatopancreas, gills, heart, gonad, muscle, and stomach) of *M. japonicus* crabs using Trizol reagent (Invitrogen, Life Technologies, Carlsbad, CA, USA) according to the manufacturer’s instructions. To remove genomic DNA contamination, the extracted RNA was treated with recombinant DNase I (RNase free) (Takara, Tokyo, Japan). We checked RNA concentration and integrity using a Nano-Drop 1000 instrument (Thermo Fisher Scientific, Carlsbad, CA, USA) followed by 0.8% agarose gel electrophoresis. cDNA was synthesized using 1 μg of RNA according to the PrimeScript™ 1st strand cDNA Synthesis Kit (Takara) protocol. After synthesis, the diluted cDNA (40-fold) was stored in a −80 °C deep-freezer. We carried out real-time RT-PCR (RT-qPCR) using Accuprep®2x Greenstar qPCR Master Mix (Bioneer, Daejeon, Korea) and Exicycler^TM^ 96 PCR machine (Bioneer). The specific primers for RT-qPCR were: *Mjp53* forward, 5′-GACAGTCATTGGGCGTCAGA-3′; *Mjp53* reverse, 5′-TTCCACAGGGTGGTGA CTCT-3′; *Catalase* forward, 5′-TGAGCCTATCGGACAGTGGA-3′; *Catalase* reverse, 5′-CCAAAGCCTTCAGATGCCG-3′; *GAPDH* forward, 5′-TGCTGATGCACCCATGTTTG-3′; *GAPDH* reverse, 5′-AGGCCCTGGACAATC TCAAAG-3′. The PCR product sizes of the *p53* and *catalase* were 127 bp and 120 bp, respectively. An internal reference was the *GAPDH* gene (147 bp). PCR thermal cycling was programmed as follows: 95 °C for 1 m, followed by 38 cycles of 95 °C for 10 s, 57 °C for 30 s, and 72 °C for 40 s. The Exicycler^TM^ 96 real-time system program (v.3.54.8) was used for verification of the RT-qPCR baseline. The relative expression levels of *p53* were calculated according to the 2^−ΔΔCt^ method.

### 2.4. Data Analysis

Statistical analysis was conducted using the Statistical Package for the Social Sciences (SPSS) v.12.0 KO (SPSS Inc., Chicago, IL, USA). All data are presented as means ± standard deviation. We performed normality and homogeneity of variances using the Levene’s test and Kruskal–Wallis test before analysis of variance (ANOVA). Two-way ANOVA was performed to determine the statistical significance of the exposure period and HBCD concentration on *Mjp53* mRNA expression. Statistically significant differences are indicated as * *p* < 0.05 and ** *p* < 0.01.

## 3. Results

### 3.1. Identification and Phylogenetic Analysis of Mjp53 Gene 

**Figure 1:**
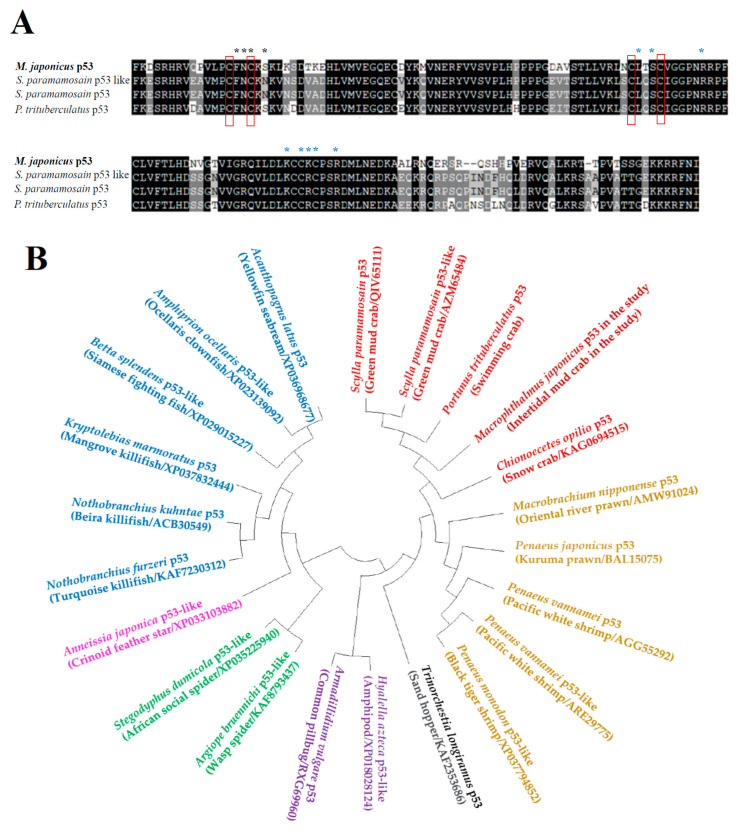
Characterization of *Macrophthalmus japonicus p53* gene. (**A**) ClustalW multiple-sequence alignment of the deduced *Mjp53* gene sequence with homologous *p53* genes of various crabs. Shaded marks in black indicated completely conserved residues in all species. Dimerization site (polypeptide-binding site) and zinc-binding site (ion-binding site) are indicated by black asterisk mark and blue asterisk mark, respectively. DNA-binding site (nucleic-acid-binding site) is presented as a red rectangular box. (**B**) Phylogenetic circle tree of *Mjp53* gene with other *p53s*. Neighbor-joining analysis showed a circle tree for phylogenetic relationships in *p53* amino acid sequences using the MEGA v.4.0 software. Bootstrap values represent 1000 replicates.

The partial sequence data of the *Mjp53* gene were obtained from the GS-FLX transcriptome database of *M. japonicus*. *Mjp53* was 477 bp long, including an open reading frame of 159 amino acids (Figure 1A). The alignment of the *Mjp53* amino acid sequence with those of other crabs revealed that residues at functional sites, including DNA-binding sites, zinc-binding sites, and dimerization sites, are conserved (Figure 1A). This implies that the partial sequence from *M. japonicus* is a predicted *p53*. The deduced amino acid sequence of *Mjp53* was 70% and 67% homologous to that of *Scylla paramamosain* (QDO16138) and *Portunus trituberculatus* (AZM65484), respectively. At the nucleotide level, there was no similarity with *p53* or *p53*-like genes of other crabs. Phylogenetic analysis placed the *Mjp53* sequence in the same clade with *p53* homologs of other crabs (Figure 1B). In crustacean species, the *Mjp53* gene formed a cluster with *p53*-homologous genes from *S. paramamosain*, *P. trituberculatus*, and *Chionoecetes opilio*. Another clade was composed of *p53* homologs involving shrimps and prawns. Fish species formed one large clade with *p53* and *p53*-like genes of various fishes. 

### 3.2. Tissue Distribution of Mjp53 Expression

**Figure 2:**
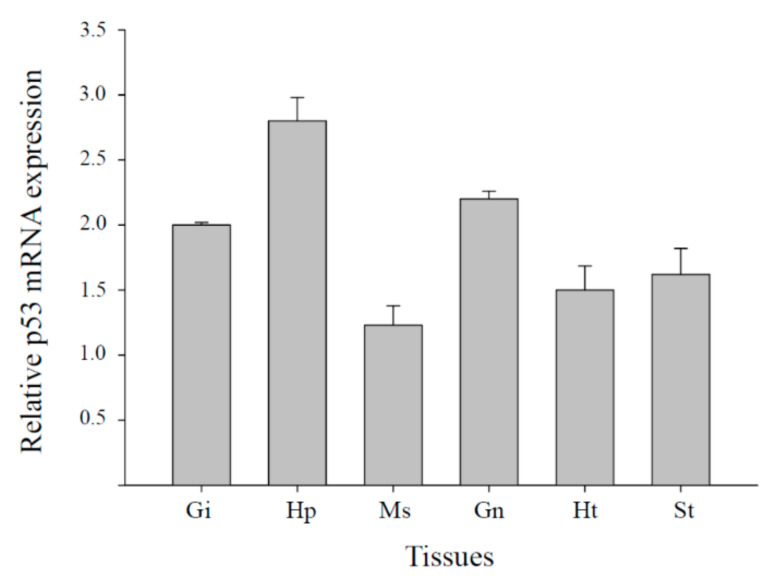
Basal transcriptional levels of *M. japonicus p53* genes in various tissues (Gi, gills; Hp, hepatopancreas; Ht, heart; Gn, gonad; Ms, muscle; and St, stomach). All data are indicated as means ± standard deviation. Expression level of *GAPDH* transcripts was used for normalization of the relative transcriptional levels in each tissue from 10 crabs. The experiment was repeated three times.

To investigate tissue-specific expression patterns, we measured *Mjp53* mRNA expression in six tissues (gills, hepatopancreas, muscle, gonad, heart, and stomach) using real-time RT-qPCR. As shown in Figure 2, high levels of *Mjp53* gene expression were observed in hepatopancreas tissues, whereas relatively low levels of *Mjp53* mRNA expression were observed in muscle. *Mjp53* gene expression was detected in all tested tissues. 

### 3.3. Catalase Gene Expression in Oxidative Stress Responses to Exposure of HBCD or BDE-47

**Figure 3:**
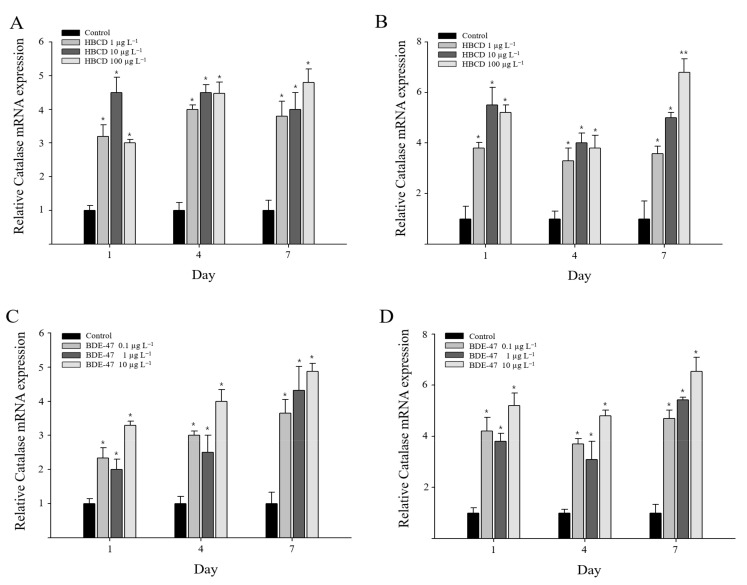
Relative transcriptional levels of *catalase* gene in *M. japonicus* gills (**A**,**C**) and hepatopancreas (**B**,**D**) after exposures to 1, 10, and 100 μg L^−1^ HBCD (**A**,**B**) and 0.1, 1, and 10 μg L^−1^ BDE-47 (**C**,**D**). Exposure periods were days 1, 4, and 7. *GAPDH* levels were used for normalization of the values. All values are indicated as mean ± SD. Statistically significant differences are presented by an asterisk mark (* *p* < 0.05 and ** *p* < 0.01) compared with the relative control value (*catalase* = 1).

*M. japonicus* catalase expression was significantly induced in the gill and hepatopancreas in response to all concentrations of HBCD or BDE-47 tested (Figure 3). After HBCD exposure, significant expression of the catalase gene (*p* < 0.05) was continuously observed from day 1 to day 7 (Figure 3A). In the hepatopancreas, on day 1, catalase mRNA expression was significantly upregulated in *M. japonicus* at all concentrations of HBCD (*p* < 0.05). The increase in catalase gene expression was decreased by day 4 compared to the level on day 1, although catalase was more upregulated in HBCD-exposed groups than in the non-exposed control group (Figure 3B). Furthermore, HBCD exposure triggered a significant induction of catalase gene expression on day 7 (*p* < 0.05) in a dose-dependent manner. Upon HBCD exposure, the highest expression of catalase was observed at a relatively high concentration of 100 µg L^−1^ HBCD (elevated 4.8-fold in gills and 6.8-fold in hepatopancreas) on day 7.

After BDE-47 exposure, the significant upregulation of catalase gene expression was observed in *M. japonicus* gill tissue (Figure 3C). Catalase gene expression patterns in response to the range of HBCD concentrations were similar on day 1 and day 4. A significant increase in catalase mRNA was identified in gill tissue for all concentrations of BDE-47 and exposure times (*p* < 0.05) (Figure 3C) in an exposure time-dependent manner. In the hepatopancreas, catalase gene expression was also significantly increased on day 1 after BDE-47 exposure (*p* < 0.05) (Figure 3D). The level of catalase gene expression decreased slightly on day 4. The expression of catalase was significantly induced on day 7 in a dose-dependent manner. The U-shaped pattern of catalase gene expression was only observed in the hepatopancreas of *M. japonicus* exposed to both HBCD and BDE-47 POPs. The highest expression of catalase was observed at the relatively high concentration of 10 µg L^−1^ BDE-47 on day 7 in gills (4.9-fold) and the hepatopancreas (6.5-fold) (*p* < 0.05). 

### 3.4. Mjp53 Gene Expression Responses to HBCD or BDE-47 Exposure

**Figure 4:**
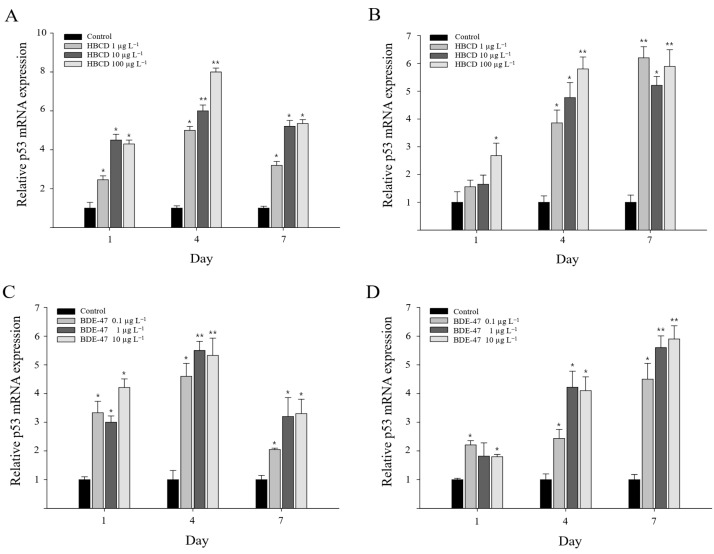
Relative transcriptional levels of *p53* gene in *M. japonicus* gills (**A**,**C**) and hepatopancreas (**B**,**D**) after exposures to 1, 10, and 100 μg L^−1^ HBCD (**A**,**B**) and 0.1, 1, and 10 μg L^−1^ BDE-47 (**C**,**D**). Exposure periods were days 1, 4, and 7. *GAPDH* levels were used for normalization of the values. All values are indicated as mean ± SD. Statistically significant differences are presented by an asterisk mark (* *p* < 0.05 and ** *p* < 0.01) compared with the relative control value (*p53* = 1).

*Mjp53* expression in response to POP (HBCD and BDE-47) exposure was also studied in gills and the hepatopancreas. On day 1, *Mjp53* expression was significantly increased in gills after HBCD exposure (*p* < 0.05) (Figure 4A). A significant increase in *Mjp53* gene expression was induced in gill tissue by exposure to all concentrations of HBCD on day 4 (*p* < 0.01). Its expression pattern was dose-dependent. *Mjp53* expression continuously increased in the gills of *M. japonicus* crabs exposed to HBCD, although expressional levels were lower on day 7 than on day 4. In the hepatopancreas, the expression of *Mjp53* mRNA on day 4 was significantly upregulated at all concentrations of HBCD in a dose-dependent manner, whereas *Mjp53* gene expression was only increased at the high concentration of 100 µg L^−1^ HBCD on day 1 (Figure 4B). By day 7, the upregulation of *Mjp53* was observed to be higher in the hepatopancreas than in gills. On HBCD exposure, the highest expression of *Mjp53* was found after exposure to 100 µg L^−1^ HBCD (8.1-fold) on day 4 in gills and 1 µg L^−1^ HBCD (6.2-fold) on day 7 in the hepatopancreas (*p* < 0.01).

BDE-47 exposure also induced the upregulation of *Mjp53* transcripts in gills and hepatopancreas of *M. japonicus* crabs (Figure 4C,D). On day 1, *Mjp53* gene expression was significantly elevated in gills after exposure to all concentrations of BDE-47. A statistically significant increase in *Mjp53* mRNA abundance was identified in gill tissue at all concentrations of BDE-47 and for all exposure times (*p* < 0.05) (Figure 4C). The highest expression of *Mjp53* mRNA was observed at the relatively high concentrations of 1 and 10 µg L^−1^ BDE-47 on day 4 in gills. In the hepatopancreas, a significant induction of *Mjp53* gene expression was observed with 0.1 and 10 µg L^−1^ BDE-47 on day 1. *Mjp53* gene expression continuously increased through days 4 and 7 after BDE-47 exposure. The increase in *Mjp53* transcript levels on day 7 was dose-dependent. The highest *Mjp53* gene expression was found at the relatively high concentration of 10 µg L^−1^ BDE-47 (5.9-fold) on day 7 in the hepatopancreas (*p* < 0.01). The POP-exposed groups exhibited significant increases in their *Mjp53* expression, indicating that the *Mjp53* gene may be involved in POP-induced apoptotic responses.

## 4. Discussion

Due to their high flame retardant efficiency and good thermal stability, HBCD and BDE-47 are widely used in polystyrene foam and textiles [1,2,23]. POPs such as HBCD and BDE-47 continue to threaten aquatic environments and cause concern as serious global pollutants [2]. However, risk assessments are restricted by poor knowledge of the distribution and quantity of these substances in aquatic environments [24]. BDE-47 toxicity induces major perturbances in terms of the reproductive, immune, and neuronal systems of aquatic organisms [25,26]. The presence of HBCD also causes oxidative damage to the thyroid system, impacts neurodevelopment, and disrupts the endocrine system [1,20,27]. However, there are insufficient studies surrounding the potential effects of POPs such as HBCD and BDE-47 in *p53*-related apoptotic responses of marine invertebrates, including *M. japonicus* mud crabs. 

In the present study, we provided supporting evidence for functional defense by apoptotic responses to HBCD or BDE-47 toxicity through the upregulation of *Mjp53* transcription. *M. japonicus*, an intertidal mud crab, is an indicator species which is able to reflect sediment toxicity conditions through POP accumulation in marine environments. Exposure to POPs such as HBCD and BDE-47 significantly increased transcription levels of *p53* in gills and hepatopancreas over all exposure periods. These results represent the first report of the induction of apoptosis through activated *p53* transcript signaling in response to HBCD and BDE-47 toxicity in crabs, although there are some limited data regarding apoptotic responses induced by increasing *p53* levels in crabs after exposure to ultraviolet radiation, *Vibrio* infection, and nitrites [28,29,30,31]. Our results are consistent with those concerning *M. japonicus* crabs in a study that indicated HBCD exposure causes oxidative DNA damage, thereby triggering the activation of *p53* transcription in the marine copepod *T. japonicus* and medaka fish (*O. melastigma*) [15,20]. In addition, BDE-47 induces oxidative-stress-mediated DNA damage with the transcriptional regulation of the apoptosis-related *p53* gene, resulting in developmental retardation in *T. japonicus* [3]. The tumor suppressor protein *p53*, as a “Guardian of the Genome”, plays an important role in cell cycle arrest, DNA repair, apoptosis, and genetic stability [12,31]. Concerning *p53* gene functions, elevated *p53* transcription might be related to the regulation of multiple cellular processes, including metabolism, antioxidant responses, and DNA repair, finally resulting in the modulation of development or decreased survival in *M. japonicus* crabs.

**Figure 5:**
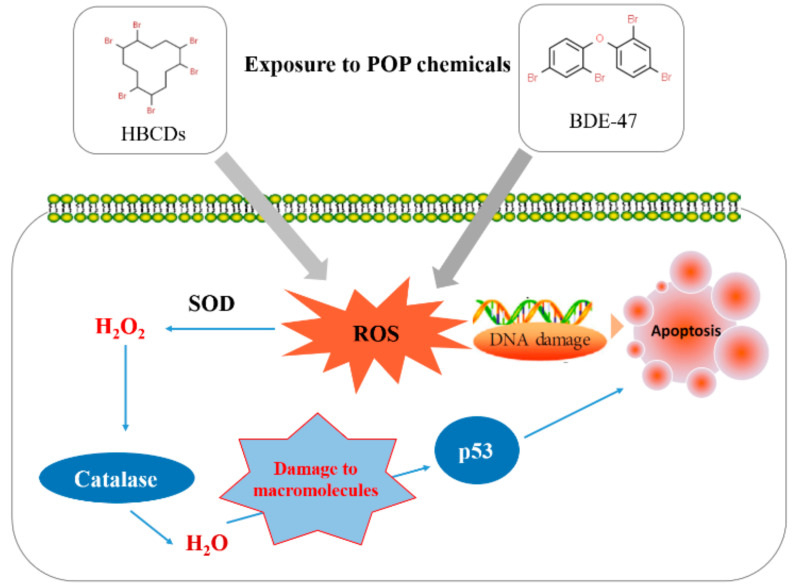
Schematic summary of the suggested molecular process involving antioxidation and *p53*-mediated apoptosis in *M. japonicus* animals exposed to POPs (HBCD and BDE-47). SOD: Superoxide dismutase, ROS: Reactive oxygen species.

The hepatopancreas is the largest organ of the digestive tract and represents the main metabolic organ in crustaceans [32]. It serves for the absorption and metabolization of nutrients, storage of energy and minerals, detoxification, digestive functions, oxygen transport, and in immune defense [32,33]. In our study, the basal levels of *p53* transcription were highest in *M. japonicus* hepatopancreas. *p53* gene expression is also high in hepatopancreas tissue of the Chinese mitten crab *Eriocheir sinensis* [12]. Our results indicated different response patterns involving *Mjp53* gene expression to HBCD and BDE-47 toxicity in gills and hepatopancreas tissue. After HBCD and BDE-47 exposure, significant *p53* gene expression changes were observed with similar patterns on day 4 in both tested tissues. However, *p53* transcriptional levels were significantly more upregulated in the hepatopancreas compared to gills exposed to HBCD or BDE-47 during long-term exposure on day 7. Environmental pollutants induce detoxification mechanisms or engage long-lasting functions involving cellular protection against antioxidants and for detoxification to diminish oxidative stress induced by exposure to POPs in the mud crab *M. japonicus* (Figure 5).

## 5. Conclusions

In this study, we provided transcriptional responses of *p53* and *catalase* genes in *M. japonicus* crabs exposed to POPs such as HBCD or BDE-47. We characterized a partial sequence of the *Mjp53* involving protein dimerization and DNA- and zinc-binding sites. The basal expression level of the *p53* gene was high in the hepatopancreas among six tissues. HBCD or BDE-47 exposures induced an increase in antioxidant catalase gene expressions in the gills and hepatopancreas of *M. japonicus*. The highest expressions of *p53* and *catalase* genes were generally observed at the relatively high concentrations of 100 µg L^−1^ HBCD and 10 µg L^−1^ BDE-47 on day 7. The BDE-47 exposure in gills was correlated with a linear increase in the *catalase* gene expression. In addition, the BDE-47 exposure in the hepatopancreas was correlated with a linear increase in the *p53* gene expression in an exposure time-dependent manner. The result indicated the induction of oxidative stress via ROS production to POP exposure. The POPs-induced oxidative stress finally boosted apoptosis responses. The transcriptional level of the apoptotic *p53* gene was significantly increased in the hepatopancreas and gills of *M. japonicus* after exposures to HBCD or BDE-47 for all exposure periods. These results suggest that apoptosis and DNA damage via long-lasting oxidative stress are found in the gills and hepatopancreas of *M. japonicus* after exposures to POPs such as HBCD or BDE-47.

## Data Availability

The data presented in this study are available on request from the corresponding author. The data are not publicly available due to reasons of privacy.
